# Supplementary material for: Cryo-EM structure of the human histamine H1 receptor/Gq complex
Source: Nat Commun. 2021 Apr 7;12:2086. doi: 10.1038/s41467-021-22427-2 (PMC8027608; doi:10.1038/s41467-021-22427-2)
Supplement: Supplementary file 1 — Supplementary Information [file 41467_2021_22427_MOESM1_ESM.pdf]

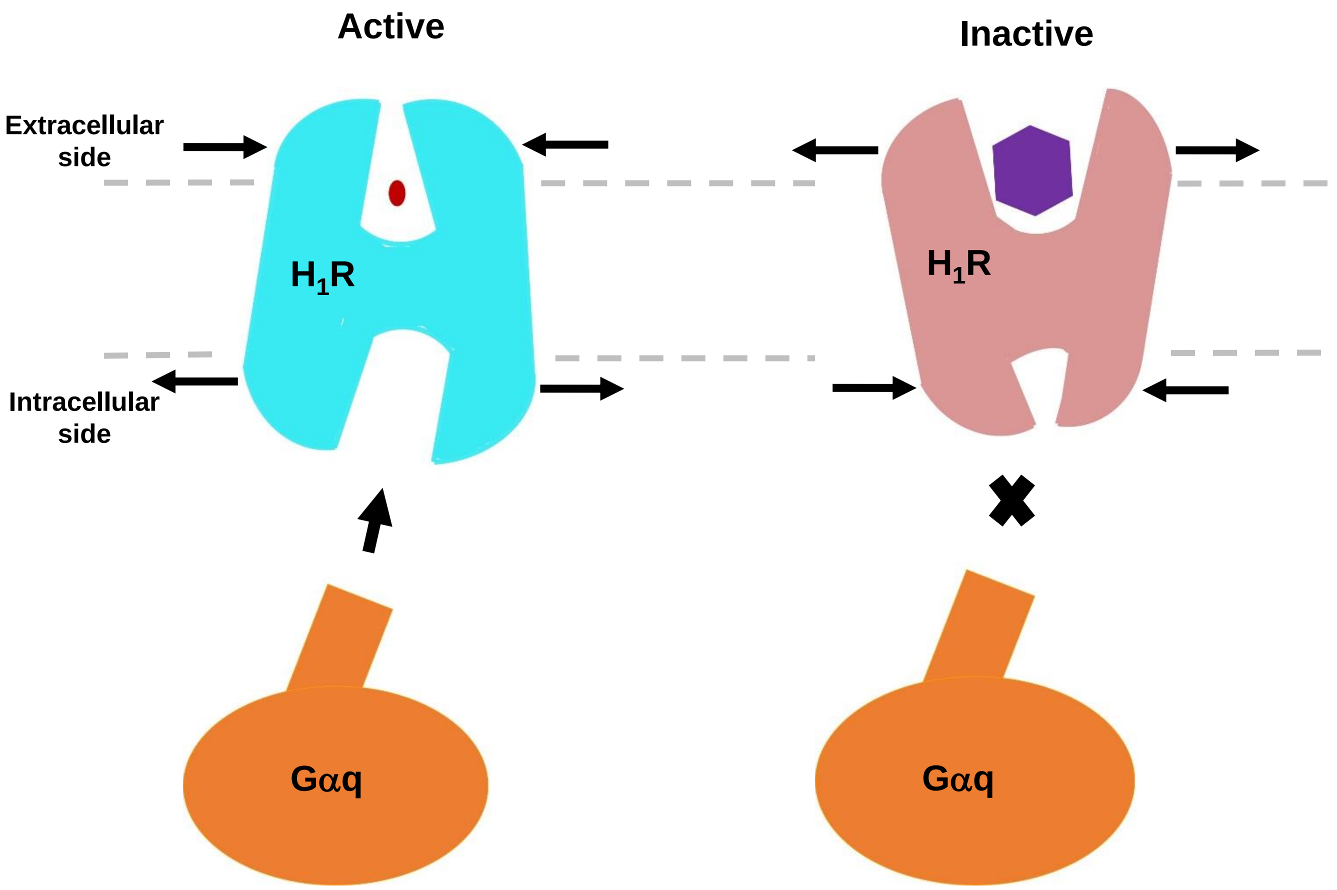

Supplementary Figure 1 | A simple scheme of the "squash to activate and expand to deactivate" model for H_1R action. Agonist (red ellipse) binding activates the receptors via squashing the ligand binding pocket on the extracellular side and opening the cavity on the intracellular side in for Gαq engagement. Conversely, antagonist (inverse agonist, purple hexagon) binding deactivates receptors via expanding the ligand binding pocket and closing the intracellular cavity to prevent Gαq engagement.

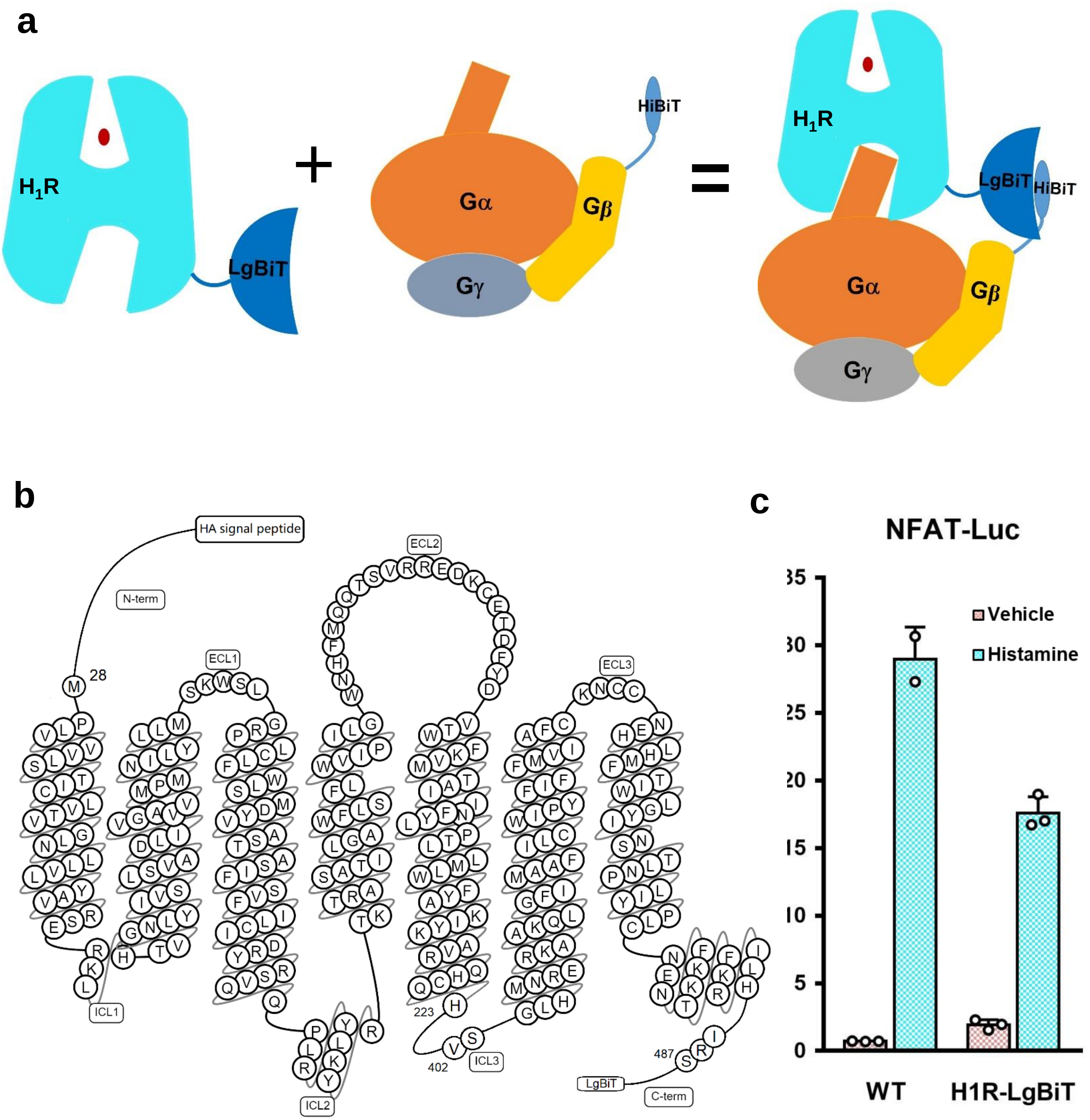

Supplementary Figure 2 | The NanoBiT tethering strategy for H_1R/Gq complex. a, A scheme of the NanoBiT tethering strategy. **b**, Snake-shape diagram of the H1R-LgBiT construct used in complex assembling, the snake-diagram of H_1R was adopted from GPCRdb. **c**, A NFAT-RE assay shows that the H1R-LgBiT retains most activity of wild type (WT) receptor. Histamine, 10µM; data are presented as mean values +/- SD; n=3 independent samples. RLU, relative luciferase unit.

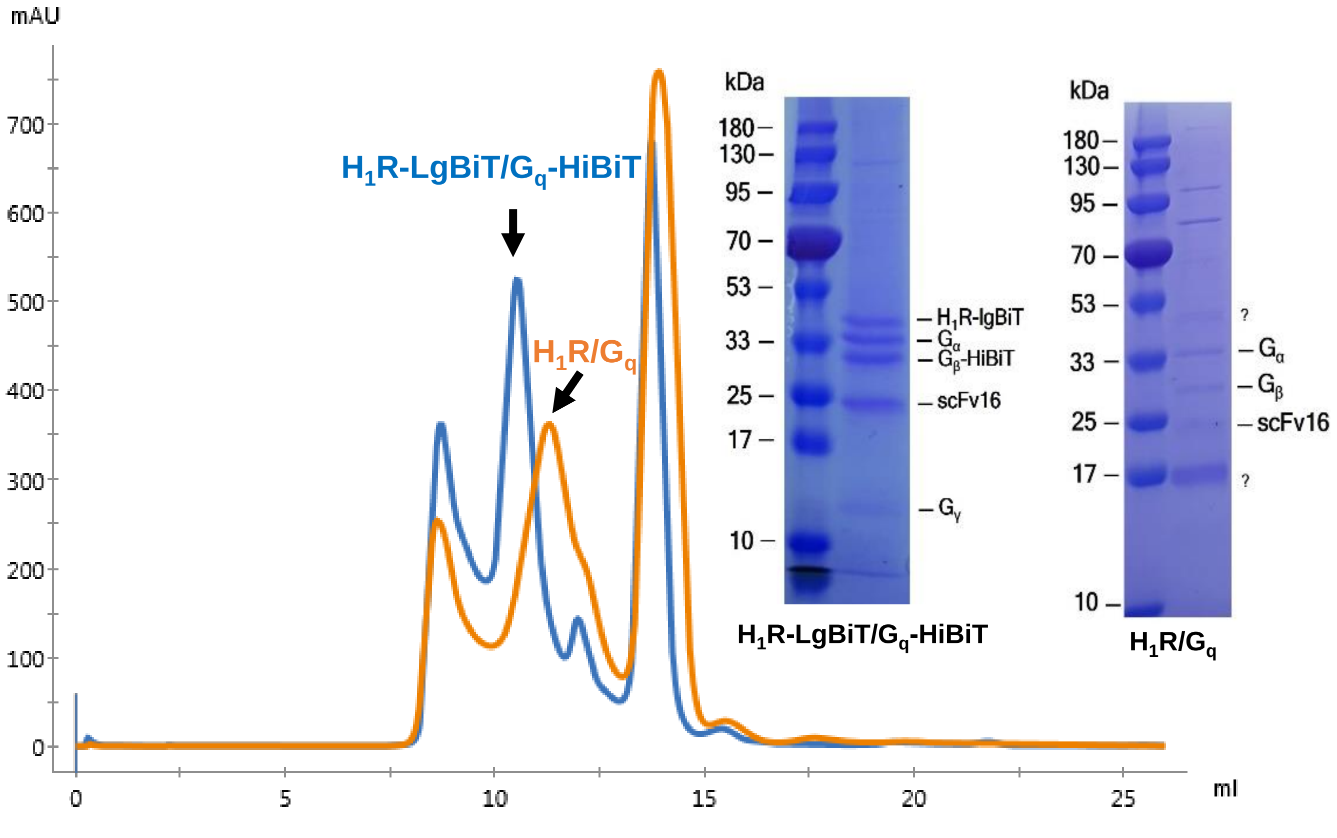

Supplementary Figure 3 | A comparison of H_1R/Gq complex purification with or without the NanoBiT strategy. The middle peak of the size-exclusion chromatography corresponds the receptor/Gq complex. The H_1R-LgBiT/Gq-HiBiT migrates to a higher molecule weight position and show a much better composition than the H_1R/Gq. The question mark "?" marks bands of unidentified proteins. The experiments was repeated independently more than two times with similar results.

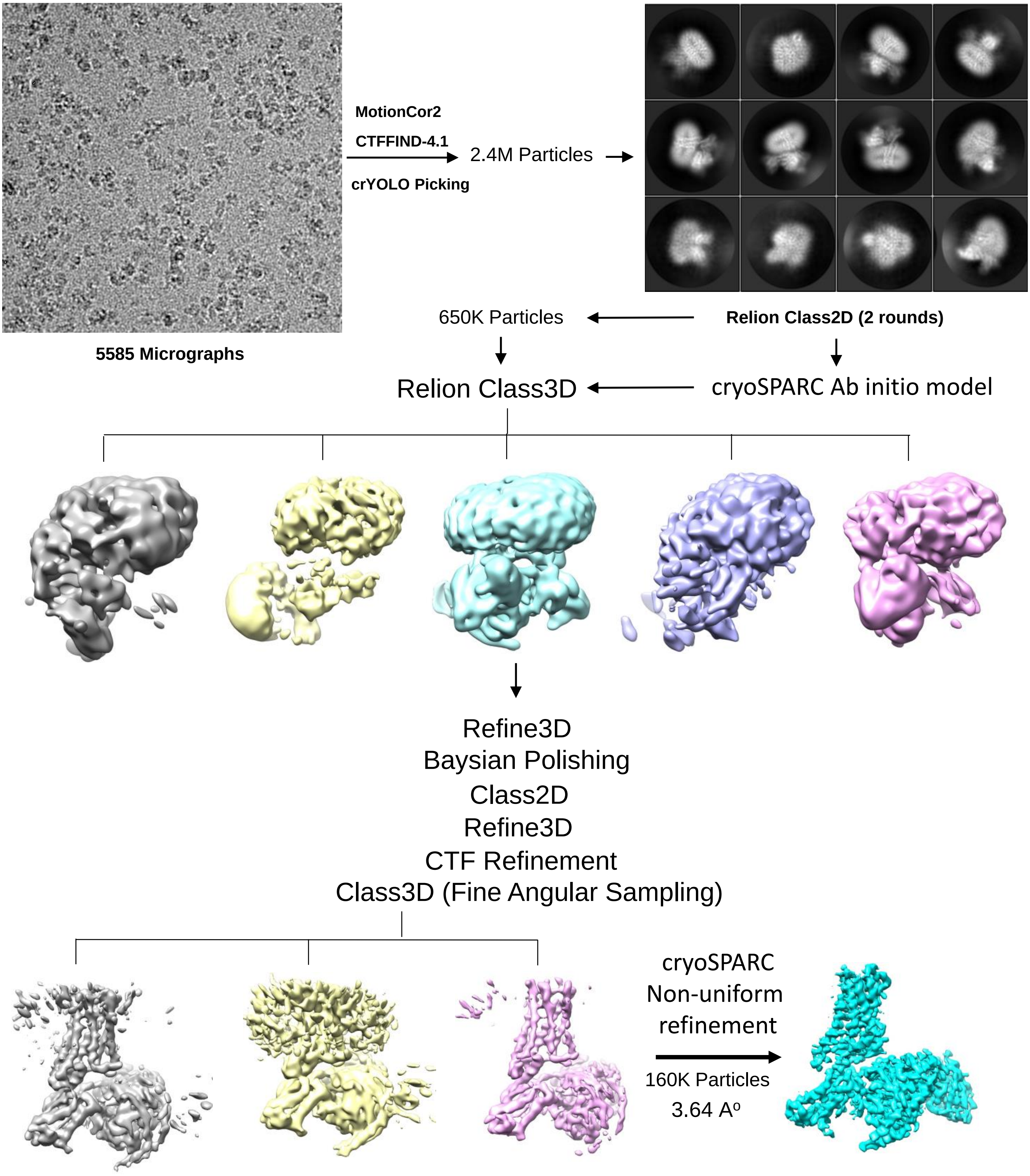

Supplementary Figure 4 | A flow-chart of the cryo-EM data process of the H_1R/Gq complex. See Methods for details.

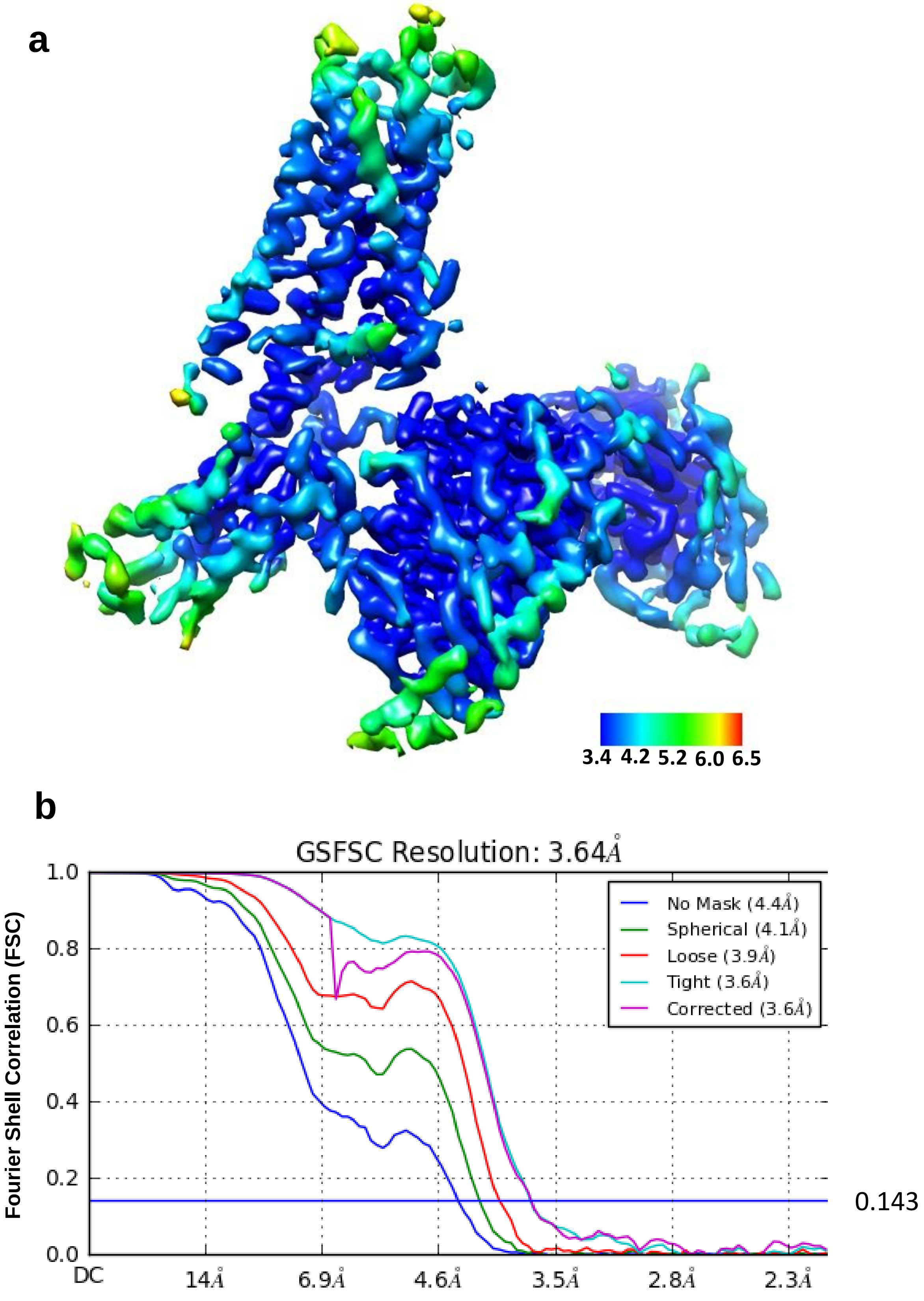

Supplementary Figure 5 | Resolution of the H_1R/G_q complex. a, Local resolution analysis of the H_1R/G_q complex. **b**, FSC curve of the H_1R/G_q complex refined by the Non-uniform refinement in cryoSPARC, the resolution was assessed by the Gold Standard of 0.143.

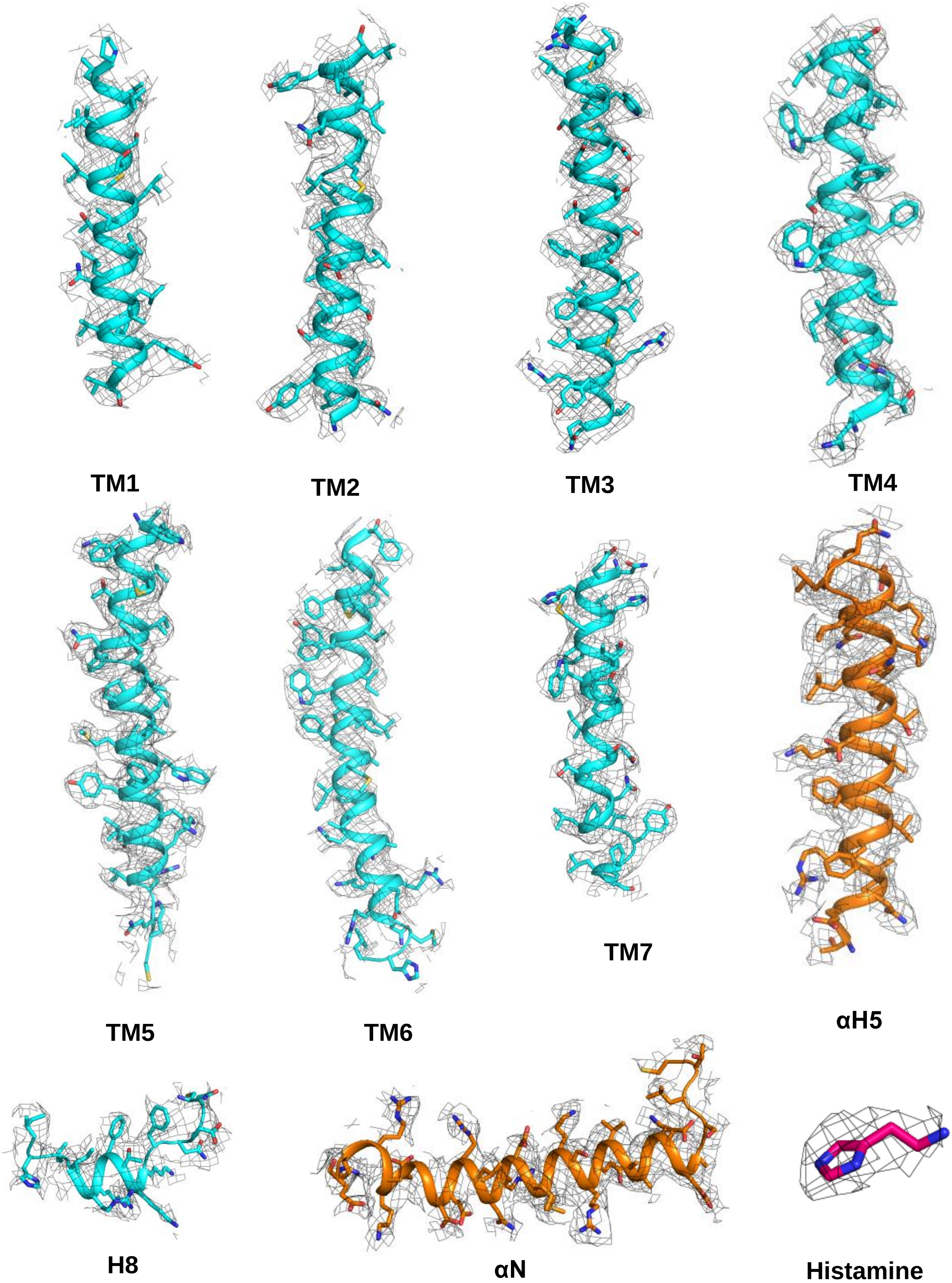

Supplementary Figure 6 | The cryo-EM density map of the representative regions of the H_1R/G_q complex. The density map was drawn by pymol at the contour level of 3.0.

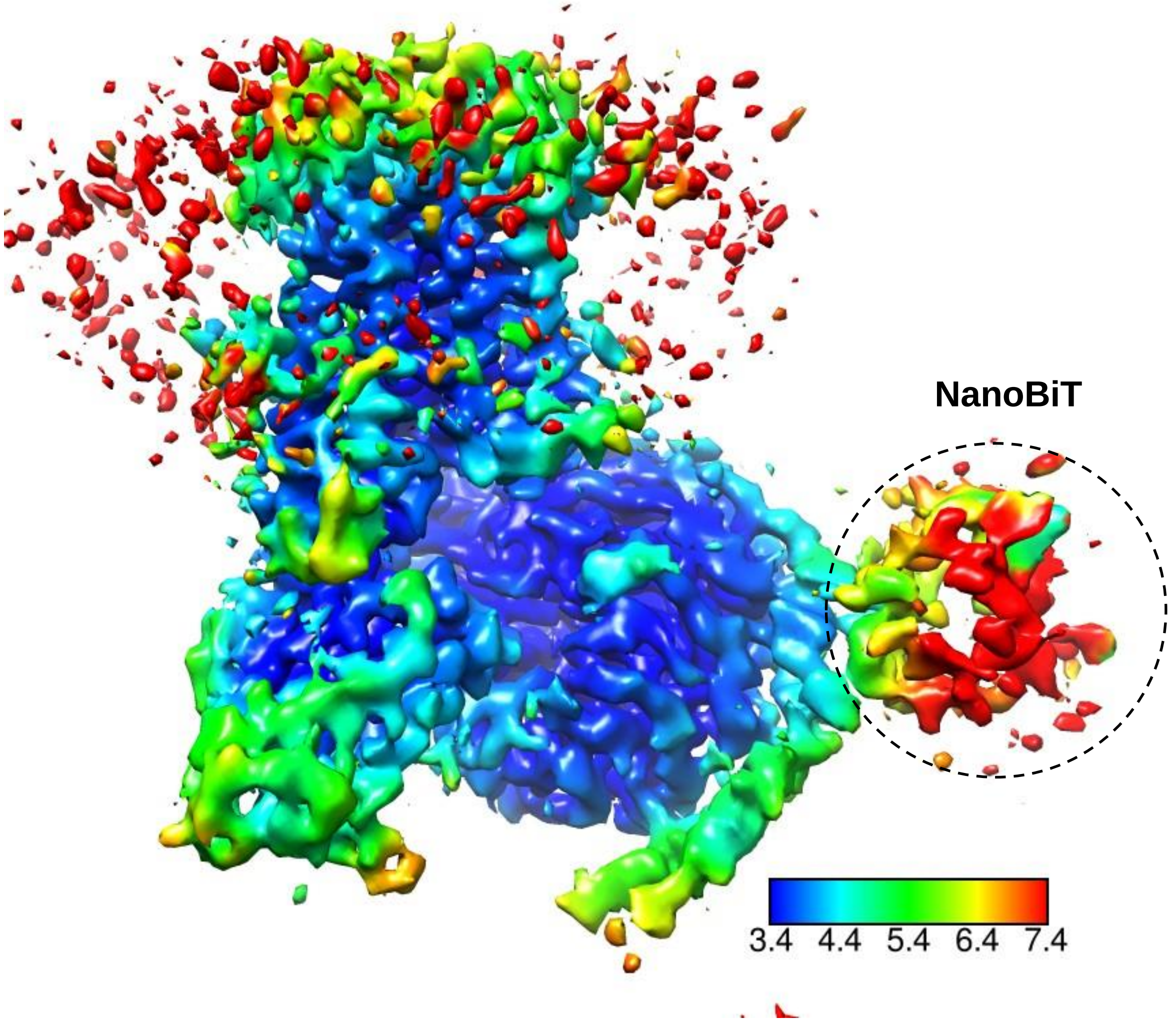

Supplementary Figure 7 | Local resolution analysis of the NanoBiT in the H_1R/G_q complex. The location of the NanoBiT were marked within the dash circle.

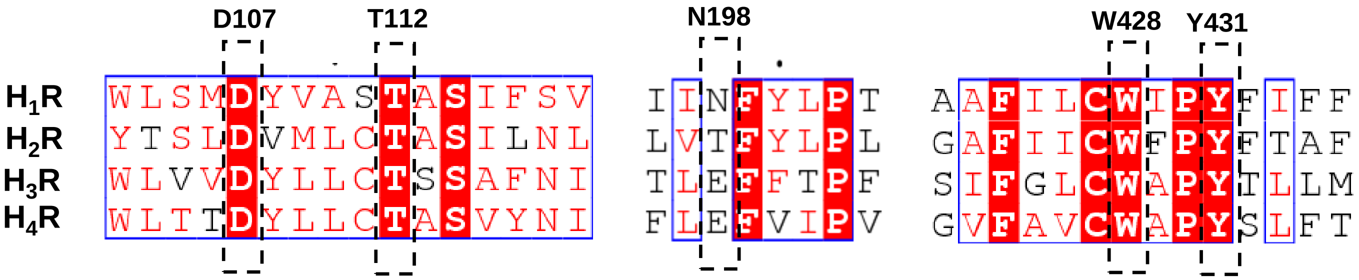

Supplementary Figure 8 | Alignment of key residues of the ligand binding pocket in the human histamine receptor family.

a

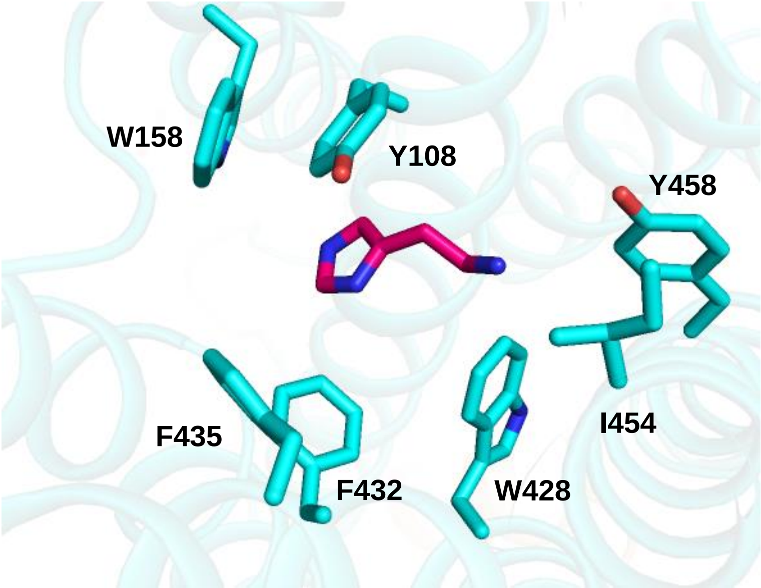

b

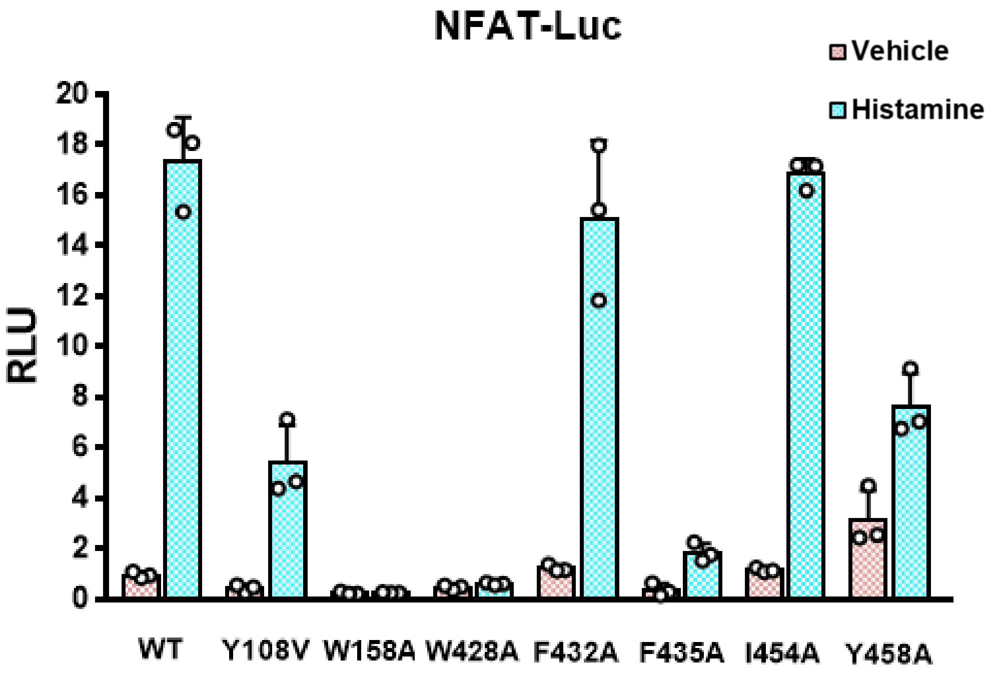

Supplementary Figure 9 | Examining the effects of non-polar residues of the ligand on receptor activation. a, The positions of non-polar residues in the ligand biding pocket of H1R**. b,** A NFAT-RE reporter assay for examining mutation of non-polar residues of the ligand binding pocket. Histamine, 10μM; data are presented as mean values +/- SD; n=3 independent samples. RLU, relative luciferase unit.

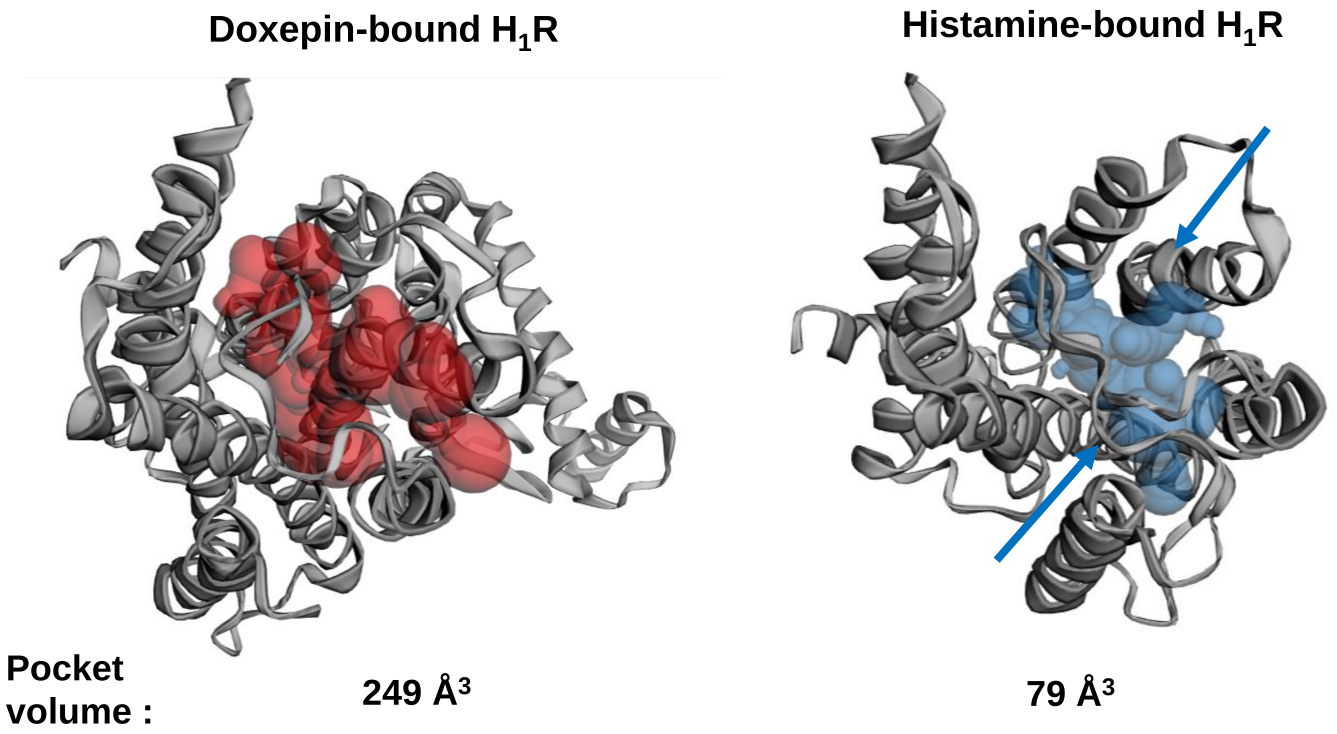

Supplementary Figure 10 | A comparison of the size of ligand binding pocket between the antagonist doxepin-bound H_1R and agonist histamine-bound H_1R. The size of the ligand binding pocket was calculated by CASTp 3.0 server. The blue arrow indicates the squashing of the pocket.

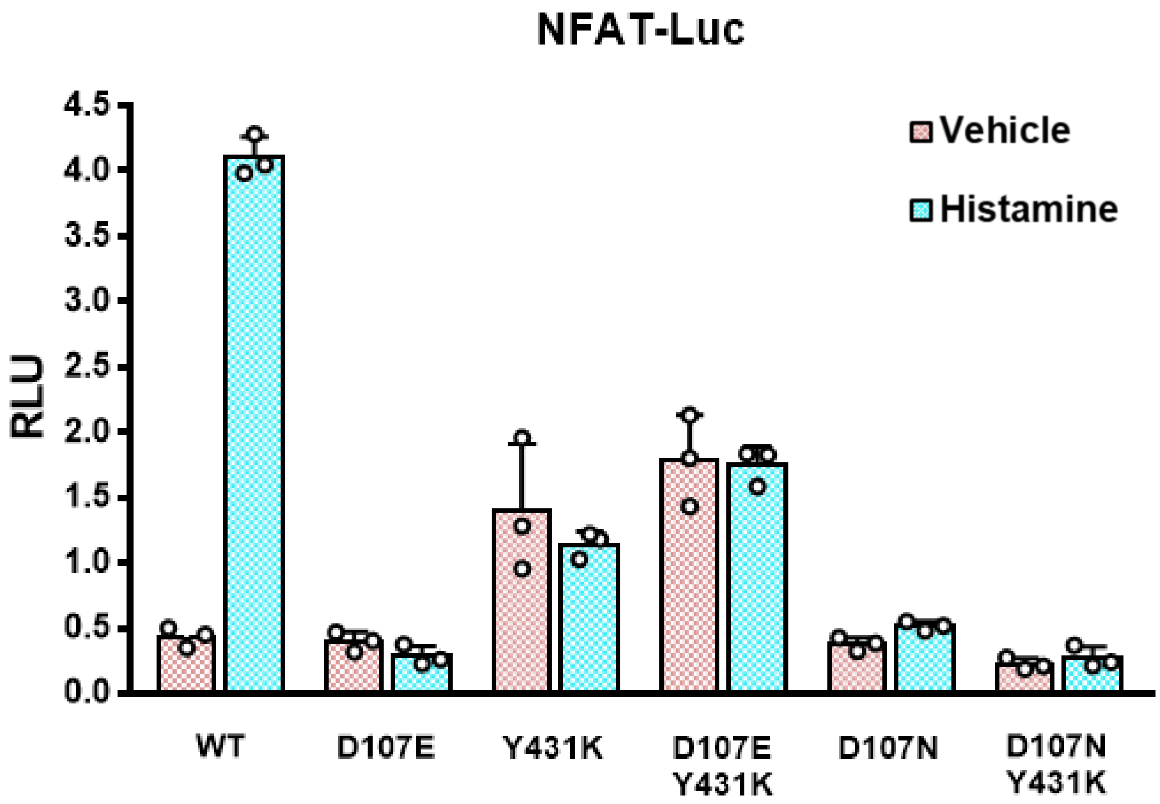

Supplementary Figure 11 | Examining the effects of key mutations of the ligand binding pocket in a NFAT-RE reporter assay. Histamine, 10µM; data are presented as mean values +/- SD; n=3 independent samples. RLU, relative luciferase unit.

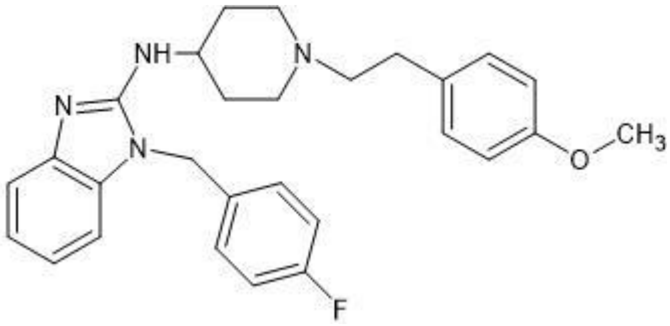

Astemizole

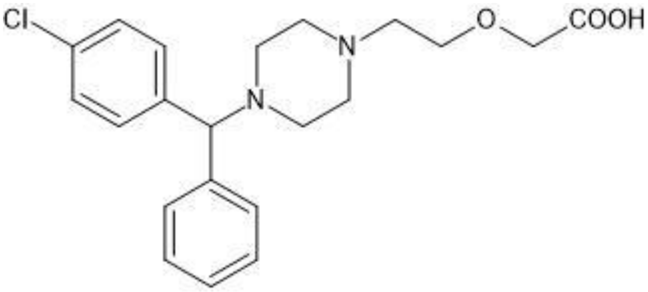

Cetirizine (Zyrtec)

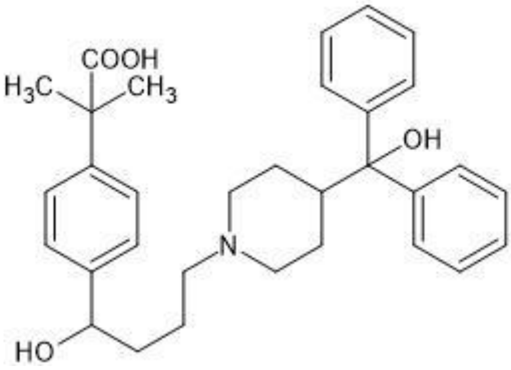

Fexofenadine (Allegra)

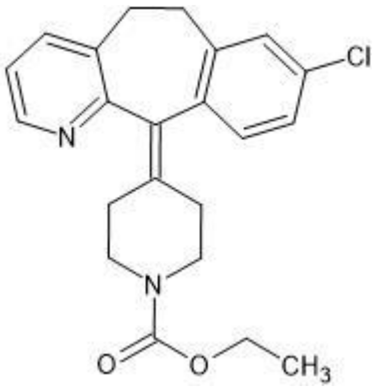

Loratidine (Claritin)

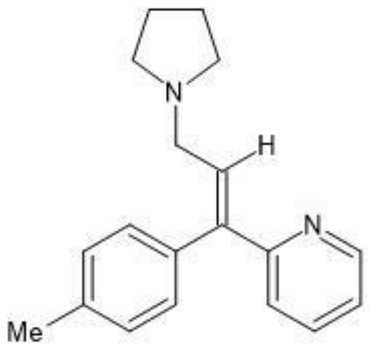

Triprolidine

Supplementary Figure 12 | Chemical structure of most commonly used antihistamines.

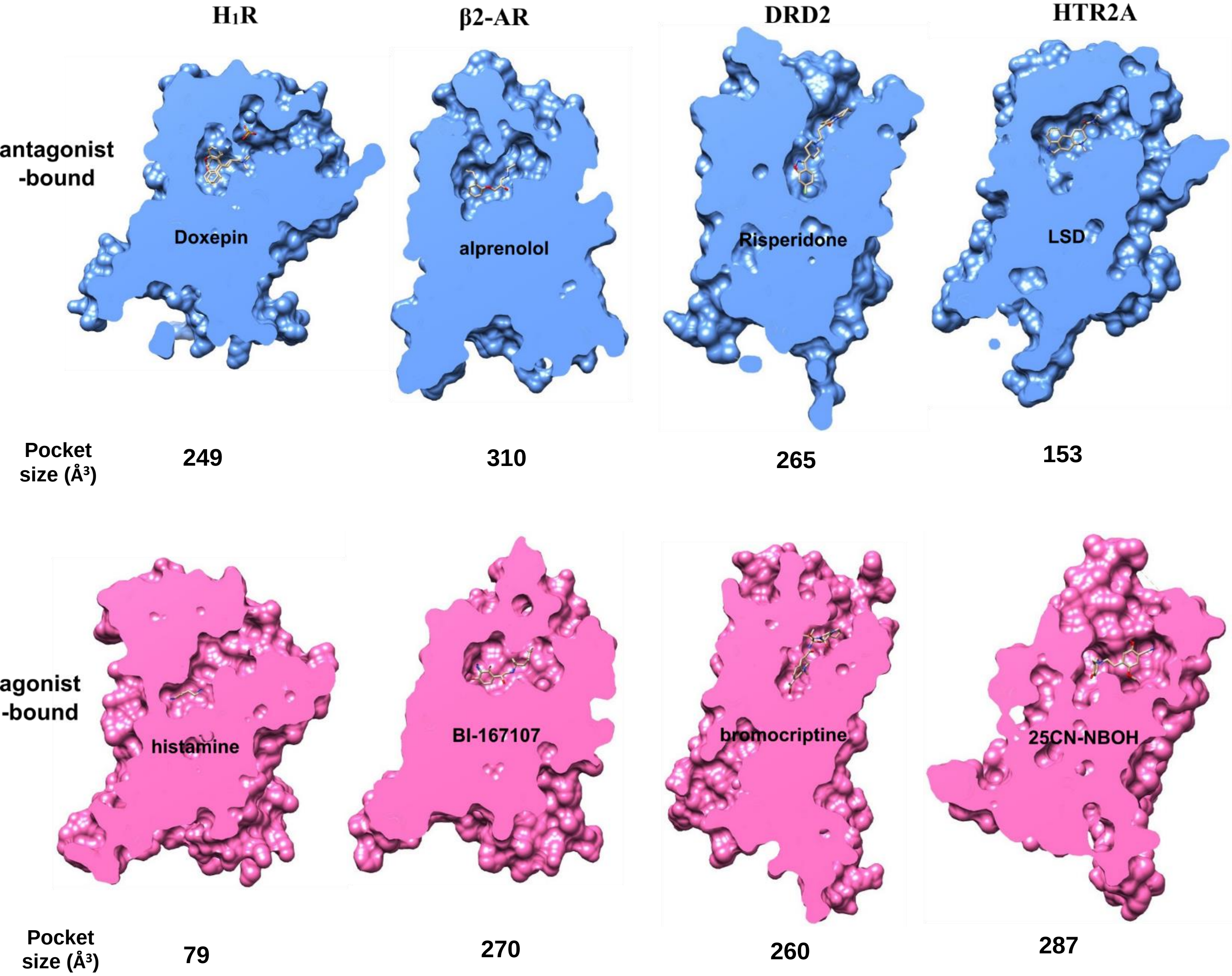

Supplementary Figure 13 | A comparison of the size of ligand binding pocket between the antagonist-bound and agonist-bound receptor among the monoamine GPCRs. The size of the ligand binding pocket was calculated by CASTp 3.0 server. The PDB code for above structures are: doxepin-bound H_1R, 3rze; alprenolol-bound β2-AR, 3nya; BI-167107-bound β2-AR, 3sn6; Riseridone-bound DRD2, 6cm4; bromocriptine-bound D2DR, 6vms; LSD-bound HTR2A, 6wgt; 25CN-NBOH-bound HTR2A, 6wha.

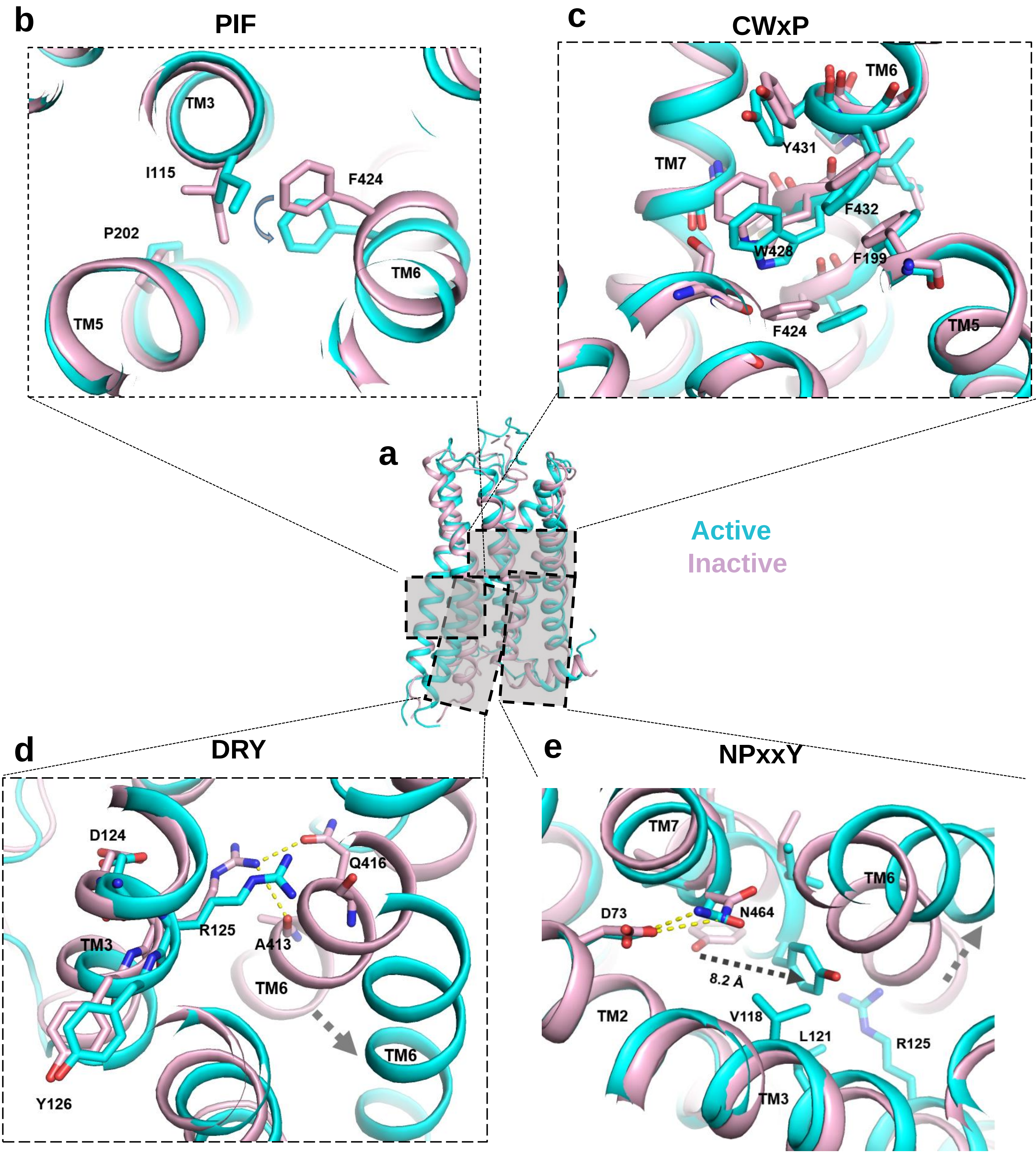

Supplementary Figure 14 | Conformational changes of the conserved motifs during receptor activation. a, Overall structure of active and inactive receptors (PDB 3RZE). **b**, The conformational changes of the PIF motif upon receptor activation. **c**, The conformational changes of the CWxP motif upon receptor activation. **d**, The conformational changes of the DRY motif upon receptor activation. **e**, The conformational changes of the NPxxY motif upon receptor activation.

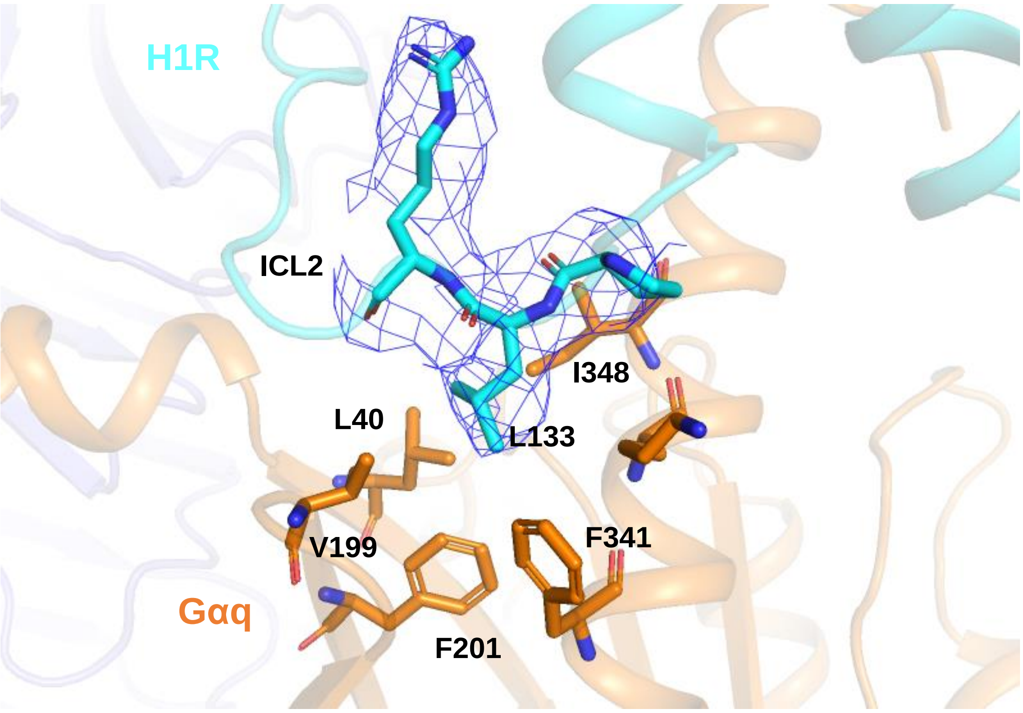

Supplementary Figure 15 | The L133 of ICL2 forms hydrophobic interaction with surrounding hydrophobic residues in the vicinity of the αN-β1 junction of Gαq. The density map (blue mesh) is set at contour level of 3.0.

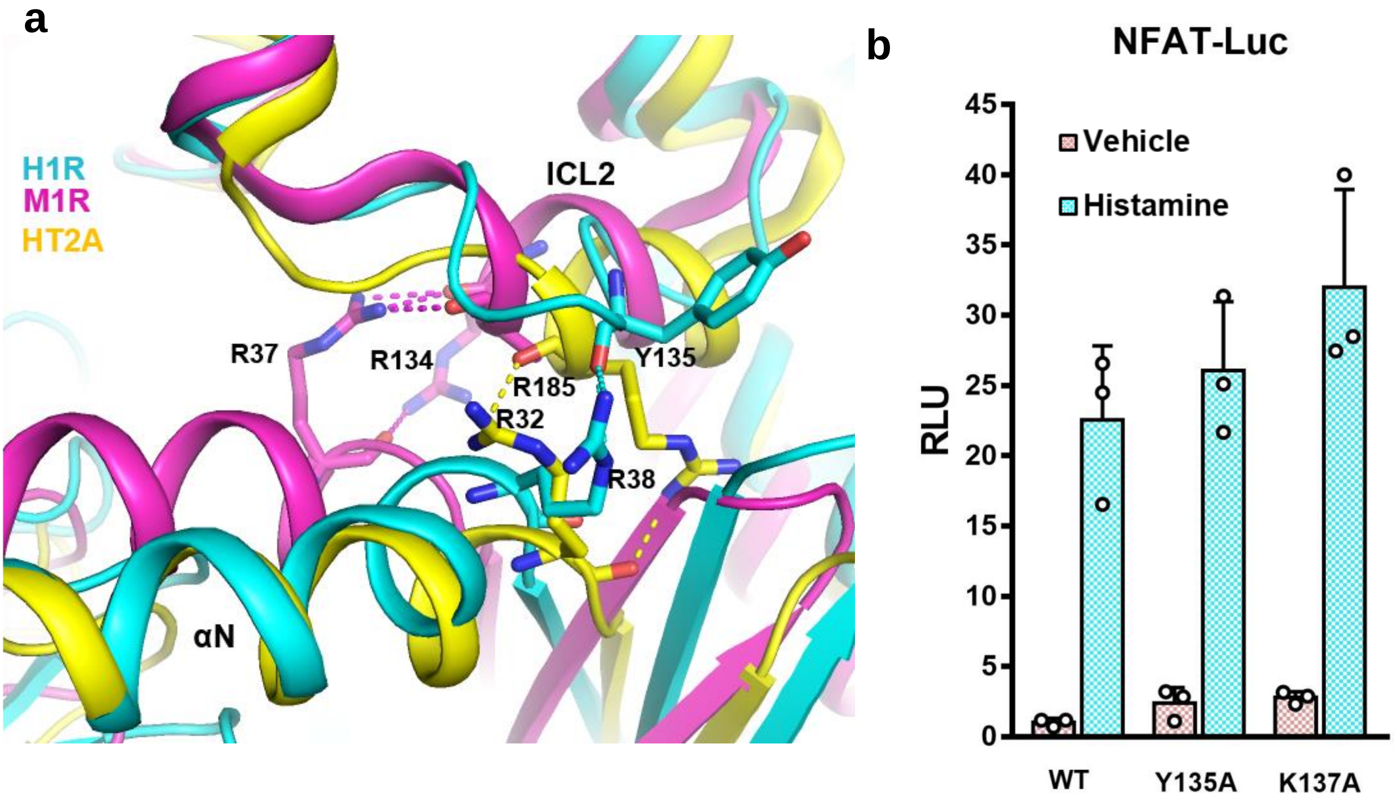

Supplementary Figure 16 | A comparison of the ICL2/αN-β1 junction interaction among the $G_{q/11}$-coupled receptors. a, The structural detail of ICL2/αN-β1 junction interaction among the $G_{q/11}$-coupled receptors. M1R/G_{11} (PDB 6OIJ), HT_{2A}/miniG_q (PDB 6WHA). **b**, The NFAT-reporter assay of H1R ICL2 mutations. Histamine, 10µM; data are presented as mean values +/- SD; n=3 independent samples.

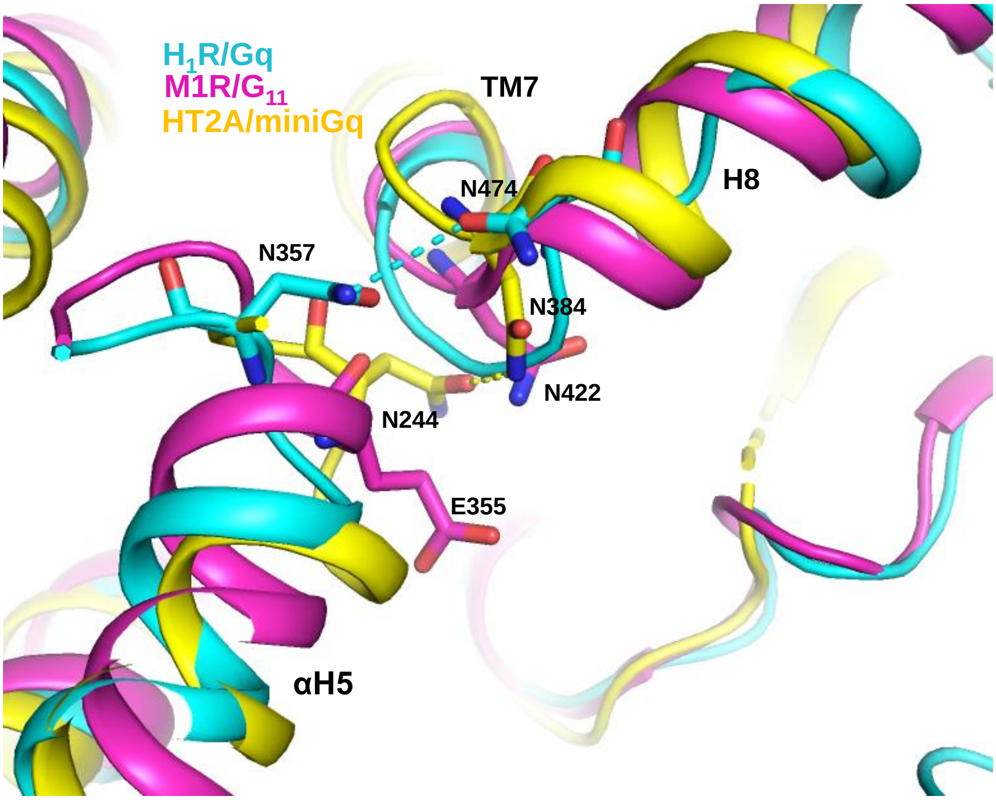

Supplementary Figure 17 | The TM7-H8 kink/αH5 interaction in the $G_{q/11}$-coupled receptors. M1R/G_{11} (PDB 6OIJ), HT_{2A}/miniG_q (PDB 6WHA).

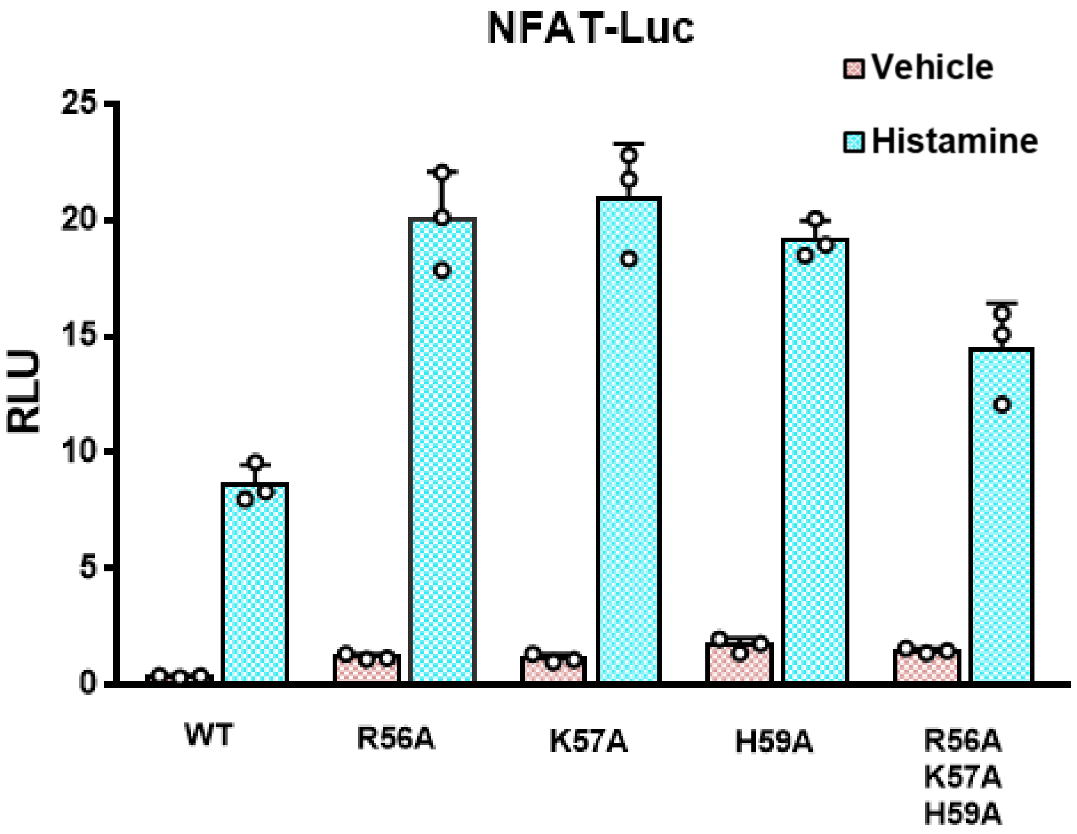

Supplementary Figure 18 | NFAT reporter assay of ICL1 mutations. Histamine, 10µM; data are presented as mean values +/- SD; n=3 independent samples. RLU, relative luciferase unit.

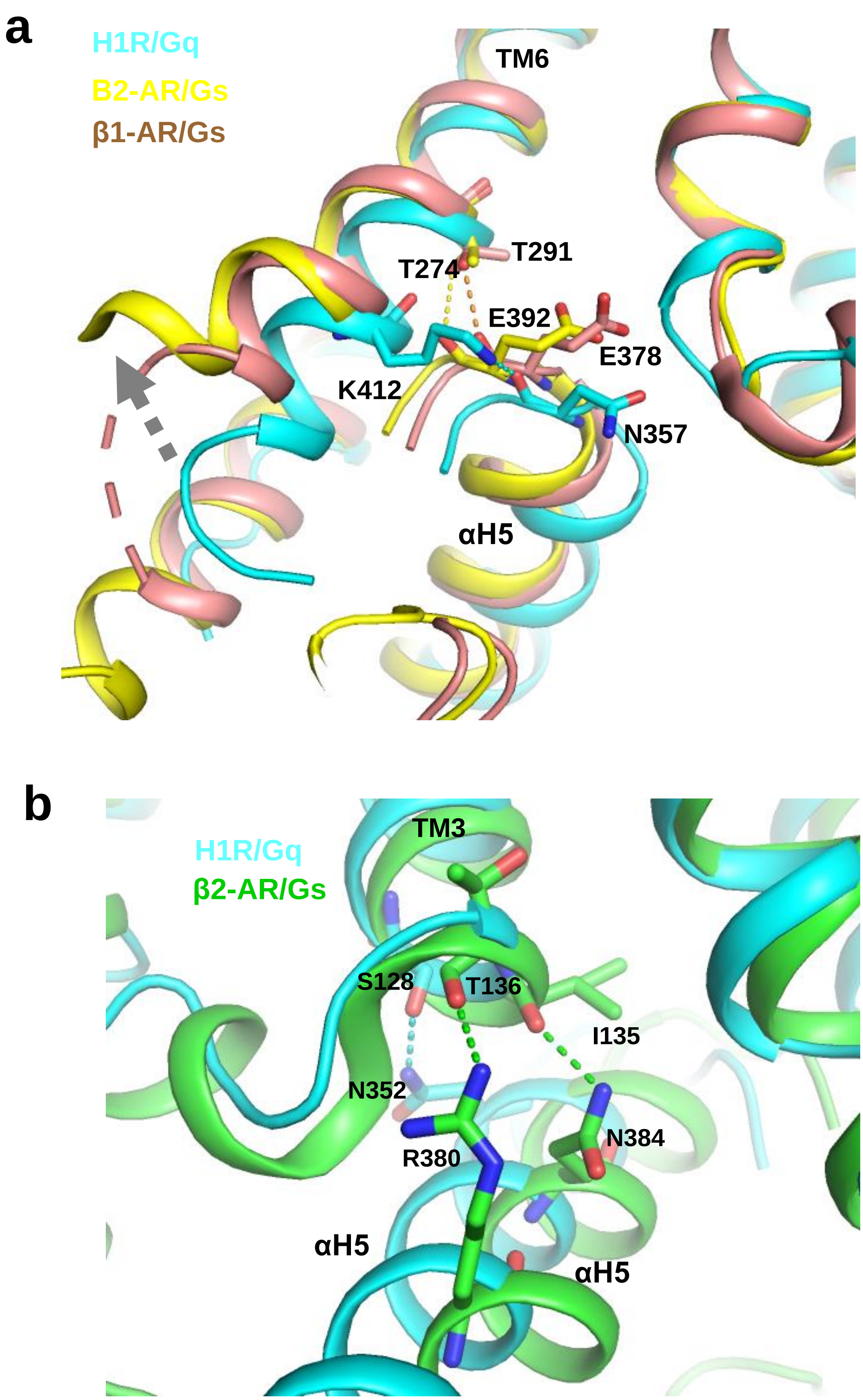

Supplementary Figure 19 | Comparisons of receptor/G-protein interaction between the class A Gs-coupled receptor and H_1R. a, The TM6-αH5 interaction. PDB code: β2-AR/Gs, 3SN6; β1-AR/Gs, 7jjo. **b,** The TM3-αH5 interaction.

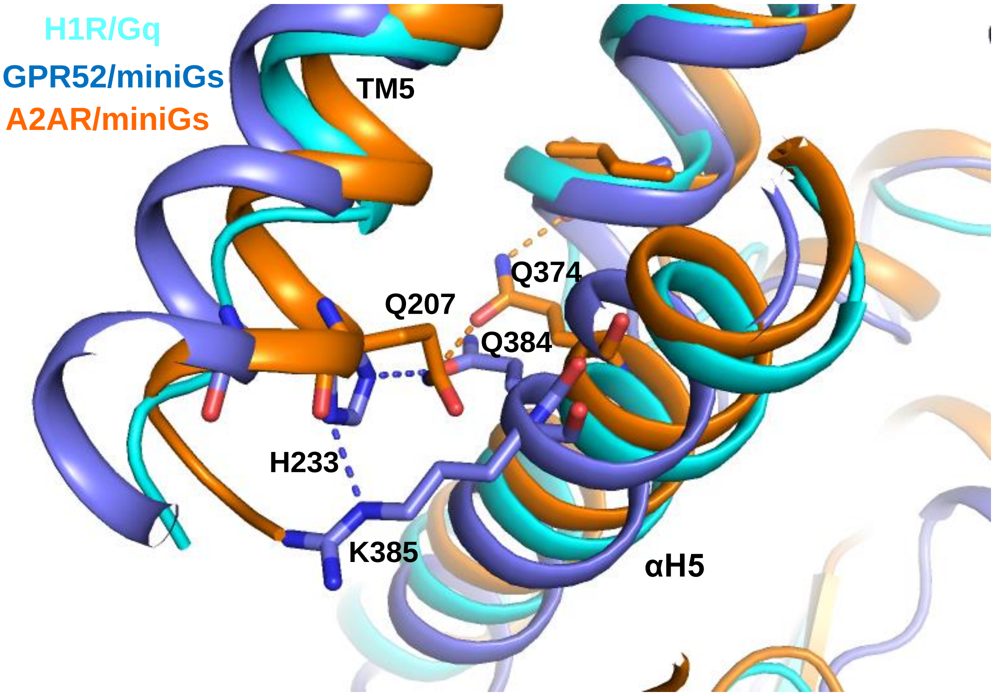

Supplementary Figure 20 | A comparison of the TM5/αH5 interaction between miniG$_s$-complexed GPCRs and G$_q$-coupled H$_1$R. PDB code: GPR52/miniGs, 3SN6; A2AR/miniGs, 6gdg.

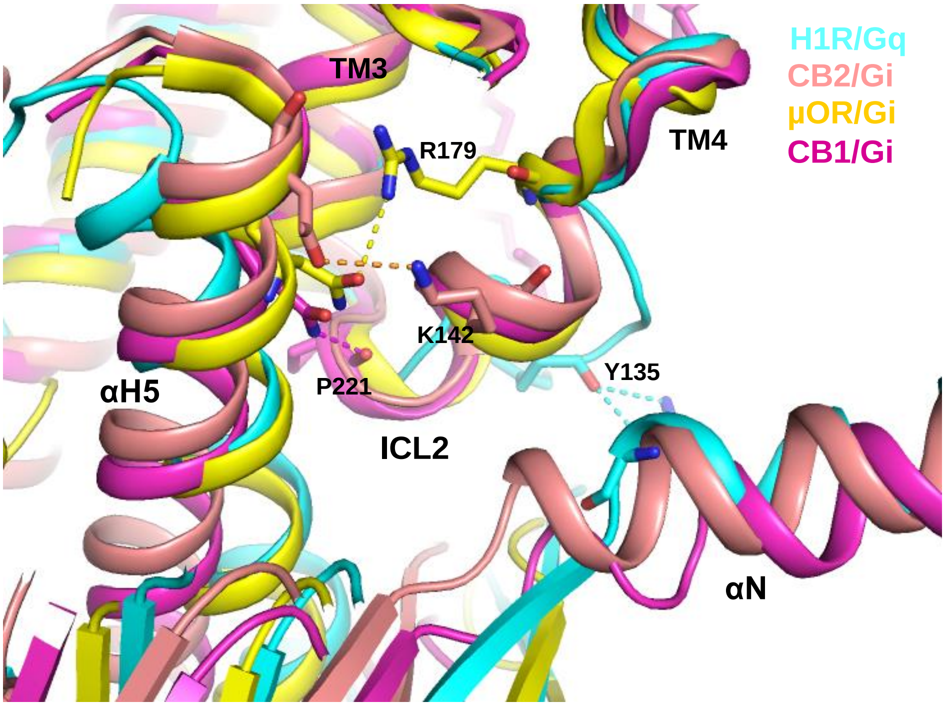

Supplementary Figure 21 | A comparison of the ICL2 interaction between G_i-coupled receptors and G_q-coupled H_1R. CB2/Gi (PDB 6PT0), μOR/Gi (PDB 6DDE), CB1/Gi (PDB 6N4B).

a

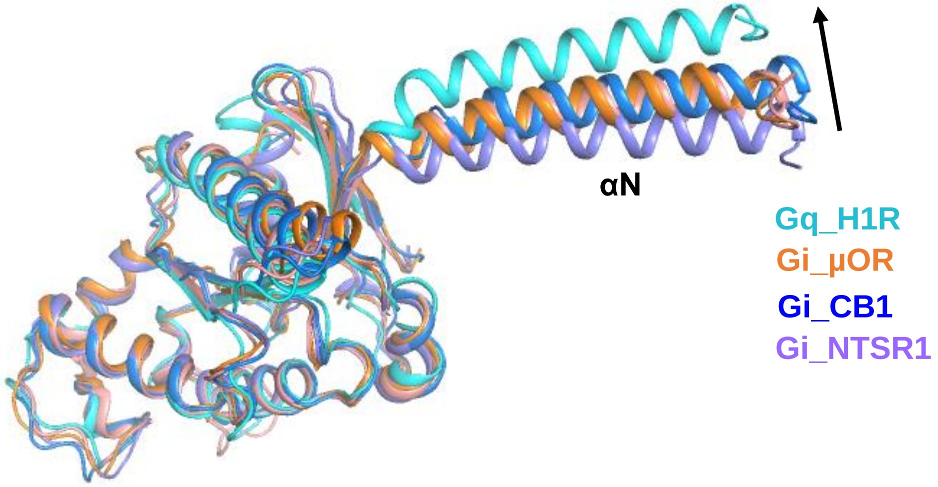

b

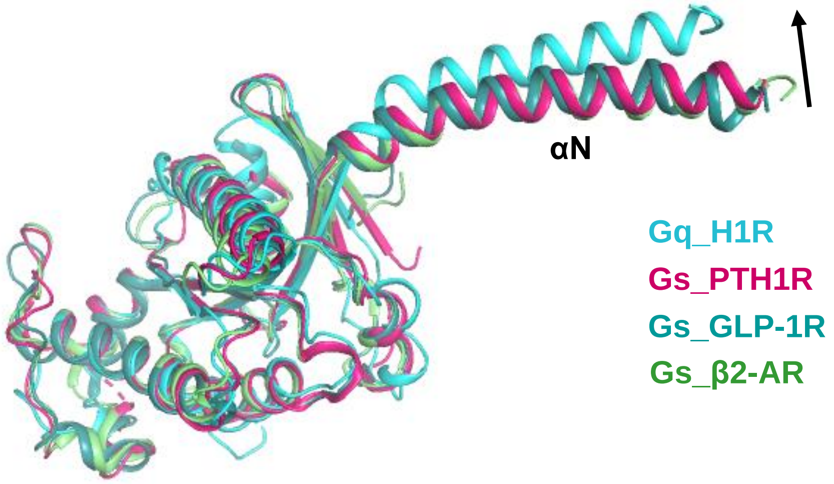

Supplementary Figure 22 | A comparison of the αN of the H_1R/Gq with aN of Gi- and Gs-coupled receptor complexes. a. A comparison with Gi-coupled receptor complex. μOR/Gi (PDB 6DDE), CB1/Gi (PDB 6N4B), NTSR/Gi (PDB 6OS9) **b,** A comparison with Gs-coupled receptor complex. PTH1R/Gs (PDB 6NBF), GLP-1R/Gs (PDB 5VAI), β2-AR/Gs (PDB 3SN6).

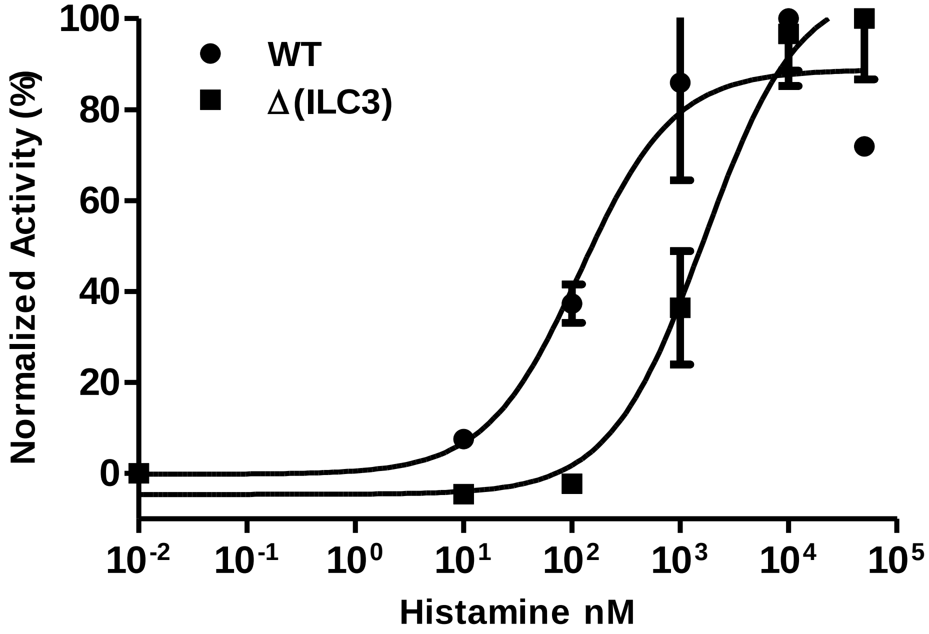

Supplementary Figure 23 | A dose response curve of H1R activation in the NFAT reporter assay. Histamine, 10μM; data are presented as mean values +/- SD; n=3 independent samples. Data were normalized to the maximal response of receptor. Δ(ICL3), the deletion of 224-401 of ICL3 of receptor. The EC50 of wild type (WT) and . Δ(ICL3) receptor are 118.6nm and 1651 nM respectively.

Supplementary Table 1| Cryo-EM data collection and refinement statistics

	H_1R/Gαqβγ/scFv16<br>EMD-30665<br>7DFL
**Data collection and processing**	
Magnification	64,000
Voltage (kV)	300
Electron exposure (e^-/Å^2)	50
Defocus range (μm)	1.2-2.0
Pixel size (Å)	0.54
Symmetry imposed	C1
Initial particle image (no.)	2,432,404
Final particle image (no.)	169,241
Map resolution (Å)	3.64
FSC threshold	0.143
**Refinement**	
Initial model used (PDB code)	3rze, 6oij
Model Resolution (Å)	3.3
FSC threshold	0.143
Map sharpening *B* factor (Å^2)	-125.1
Model composition	
Non-hydrogen atoms	8987
Protein residues	1133
Ligands	1
B factor (Å^2)	
Protein	71.61
Ligand	68.73
R.m.s. deviations	
Bond length (Å)	0.007
Bond angles (°)	1.097
Validation	
MolProbity score	1.9
Clashscore	9.45
Poor rotamers (%)	0
Ramachandran plot	
Favored (%)	94.08
Allowed (%)	5.92
Disallowed	0
